# Case report: A HIV-negative hemodialysis patient positive for pANCA with severe pneumocystis pneumonia: A case report and review of literature

**DOI:** 10.1097/MD.0000000000033351

**Published:** 2023-03-24

**Authors:** Jingda Huang, Fang Zeng, Jiajie Li, Wang Xu, Meirong Shen, Qiao Shu, Dehui Liu

**Affiliations:** a Department of Nephrology, The First Hospital of Jilin University, Changchun, Jilin, China; b Department of Nephrology, Ganzhou People’s Hospital, Ganzhou, Jiangxi, China; c Department of Hepatobiliary and Pancreatic, The First Hospital of Jilin University, Changchun, Jilin, China; d Department of Cardiovascular, Ganzhou People’s Hospital, Ganzhou, Jiangxi, China; e Department of Critical Care Medicine, Ganzhou People’s Hospital, Ganzhou, Jiangxi, China.

**Keywords:** ANCA associated vasculitis, case report, hemodialysis, immunosuppression, pneumocystis pneumonia

## Abstract

Pneumocystis pneumonia (PCP) is an opportunistic fungal infection that occurs in people with impaired or suppressed immunity such as patients with human immunodeficiency virus or organ transplant. However, the incidence and characteristics of PCP in the population with long-term hemodialysis is poorly described in the literature.

**Patient concerns:**

We present a case of a 50-year-old female patient being transferred to our hospital in February 2022 with a 20-day history of cough and tight breath. She received amoxicillin and cephalosporin anti-infection treatment successively in local hospital but no significant improvement in symptoms. She had a 2-year history of hemodialysis and no relevant transplantation and human immunodeficiency virus infection. She was diagnosed as ANCA associated vasculitis (AAV) and given oral prednisone acetate (20 mg/day) and methotrexate (2.5 mg/week) half a year ago.

**Diagnoses:**

Based on the patient’s medical history, Lung computerized tomography image, the Next generation sequencing report, the patient was diagnosed with renal failure, anti-neutrophil cytoplasmic antibody associated vasculitis, and Pneumocystis pneumonia.

**Interventions:**

The dosage of immunosuppressant was reduced due to leucocyte dripping and fever, and antibiotic and antifungal treatment were also given. The patient’s lung condition was getting worse and noninvasive ventilator was required to maintain blood oxygen. Blood filtration is used to remove toxins. Ganciclovir and trimethoprim-sulfamethoxazole was used based on the next generation sequencing report.

**Outcomes:**

The patient died of respiratory failure.

**Lessons:**

The risk of PCP in hemodialysis patients may be higher than that in ordinary population, and the prognosis of patients with immunosuppression may be worse. Dynamic assessment of vasculitis activity is necessary for hemodialysis patients with AAV because infections may obscure lung symptoms of AAV. It is not recommended that hemodialysis patients with long-term immunosuppression should reduce or stop the dosage of immunosuppressive drugs during the treatment because it may aggravate the condition of PCP. There is still no clear conclusion on whether hemodialysis patients need preventive medicine, but the identification of risk factors and early diagnosis and treatment are important for the prognosis of PCP on hemodialysis population.

## 1. Introduction

Pneumocystis jirovecii is an opportunistic yeast-like fungus best recognized for causing pneumocystis jirovecii pneumocystis pneumonia (PCP).^[1]^ Prior to the 1980s, PCP was best known as a pediatric disease, found mainly in malnourished infants.^[2]^ With human immunodeficiency virus (HIV) spreading around the world, the prevalence and awareness of pneumocystis increased significantly in HIV population. Recent observational and descriptive studies indicate that PCP is becoming a public health concern in immunocompromised patients without HIV, including patients with hematologic and solid organ malignancies, solid organ transplant recipients, and autoimmune diseases.^[3]^ Hemodialysis patients have immunological dysregulation, involving both the innate and adaptive response,^[4]^ which are at high risk of opportunistic infection. The relationship of hemodialysis with pneumocystis jiroveci pneumonia (PJP) infection has not been specifically stressed previously. There were few reports on hemodialysis patients with PCP. Here, we report a case of a HIV-negative hemodialysis patient positive for perinuclear anti-neutrophil cytoplasmic antibody (pANCA) with severe Pneumocystis pneumonia and review related literatures.

## 2. Case presentation

A 50-year-old female patient was transferred to our hospital in February 2022 with a 20-day history of cough and tight breath. She had a 2-year history of hemodialysis and no relevant transplantation and HIV infection. The patient had cough and fatigue without obvious inducement 2 years ago, and renal function examination showed that her blood creatinine was over 1000 umol/L (11.3mg/L). She received forearm arteriovenous fistulation and was treated with maintenance hemodialysis for 3 times per week until now. Half a year ago, she had cough and small amount blood in sputum accompanied by chest tightness that aggravated after activity. Chest computerized tomography (CT) showed pneumonia and blood tests showed blood creatinine of 835 umol/L (9.4 mg/L), pANCA and myeloperoxidase (MPO) positive. She was diagnosed as ANCA associated vasculitis (AAV) and bacterial pneumonia and was treated with anti-infection, hemodialysis, immunosuppression, symptomatic treatment, and discharged after symptoms improved and was given oral prednisone acetate (20 mg/day) and methotrexate (2.5 mg/week). During the course of the disease, the patient had lung infections repeatedly and received antibiotic treatment for several times. Twenty days prior, the patient coughed and coughed yellow and white phlegm after catching cold, accompanied by chest tightness, and tight breath and no fever. She received amoxicillin and cephalosporin anti-infection treatment successively in local hospital but no significant improvement in symptoms. Then she was transferred to our hospital and admitted to our department.

The physical examination showed blood pressure of 154/109 mm Hg, heart rate of 76 beats/minute, respiratory rate of 20 rates/ minute, and temperature of 36.6°C, moist rales were audible over bilateral lungs, without lower-extremity edema. Physical examination of the heart and abdomen were normal. Blood tests performed on admission showed white blood cell (WBC) count of 2.96 × 10E9/L, percentage of lymphocytes (LYM %) of 9.50%, absolute value of lymphocyte (LYMP) of 0.28 × 10E9/L, hemoglobin level of 105g/L, platelet count of 127 × 10E9/L, potassium concentration of 6.1g/L. Aspartate aminotransferase of 23.4 U/L, alanine aminotransferase of 18.1U/L, blood urea nitrogen of 27.4 mmol/L, and serum creatinine of 929.9 μmol/L (10.5 mg/L). Hypersensitive C-reactive protein was 5.7 mg/ L. Stool occult blood test was positive. pANCA titer was 1:32. cytoplasmic anti-neutrophil cytoplasmic antibody, MPO, proteinase 3 and anti-glomerular basement membrane antibody were all negative. Flow cytometry of peripheral blood cells showed CD3 + CD4 + Tcells/LYMP was 54.6 %, CD3 + CD8 + T cells/LYMP was 15.0 %, CD3 + CD4 + Tcells/CD3 + CD8 + T cells was 3.65, CD19 + B cells/LYMP was 12%. Serum 1, 3-beta-D-glucan level was 27.9 pg/mL (<10 pg/mL). Tuberculosis infection T cell spot test and antituberculosis antibody assay were negative. Bacterial endotoxin level was <0.01 EU/mL and aspergillus galactomannan was negative based on blood test. Hepatitis B and C viruses, HIV, and Syphilis were all tested negative by serology. The test of nucleic acid of corona virus disease 2019 was negative (Table 1).

**Table 1:** Laboratory data on admission.

Blood count			Immune-related	
WBC (3.50–9.50)	2.96 × 10^9^/L	IgG (860.00–1740.00)		708.00 mg/dL
RBC (3.80–5.10)	3.82 × 10^12^/L	C3 (70.00–140.00)		66.20mg/dL
HGB (115.00–150.00)	105g/L	pANCA (<1:10)		1:32
PLT (125.00–350.00)	127 × 10^9^/L	cANCA (<1:10)		Negative
NEUT% (40%–75%)	71.2%	MPO		Negative
LYMPH% (20%–50%)	9.5%	PR3		Negative
LYMPH (1.10–3.20)	0.28 × 10^9^/L	GBM		Negative
Blood chemistry		Lymphocyte CD analysis		
ALT (7–40)	18.10U/L	CD3 + Tcell (50–84)		69.6%
TBIL (0–23.00)	4.40 μmol/L	Tc (CD3 + CD8+) (15–44)		14.97%
ALB (40–55)	38.90 g/L	Th (CD3 + CD4+) (27–51)		54.61%
UREA (2.6–7.5)	27.41 mmol/L	CD3 + CD4+/CD3 + CD8 + (0.98–2.0)		3.65
CREA (41–73)	929.9 μmol/L	CD19 + Bcell (5–18)		19.64%
TG (<1.70)	1.92 mmol/L	CD16 + 56 + NKcell (8.1–40)		11.18%
K (3.5–5.3)	6.05 mmol/L			
Na (137–147)	137.83 mmol/L	Pathogen		
Cl (99–110)	99.44 mmol/L	Sputum culture		Plenty of G + cocci
Ca (2.11–2.52)	2.34 mmol/L	Fungal smear		Negative
P (0.85–1.51)	1.25mmol/L	Tuberculosis smear		Negative
CRP (<6.0)	5.73 mg/L	Bacterial endotoxin (<10)		0.20 EU/mL
PCT (<0.05)	0.27 ng/mL	Fungal glucan (<10)		16.00 pg/mL
		GM		Negative
		T-SPOT		Negative
		COVID-19 RNA		Negative

ALT = alanine aminotransferase, cANCA = cytoplasmic anti-neutrophil cytoplasmic antibody, COVID-19 = corona virus disease 2019, CRP = C-reactive protein, LYMP = lymphocyte, LYMP = lymphocyte, MPO = myeloperoxidase, pANCA = perinuclear anti-neutrophil cytoplasmic antibody, PCT = procalcitonin, PR3 = proteinase 3, Th = T helper, T-SPOT = Tuberculosis infection T cell spot test, WBC = white blood cell.

**Figure 1.**
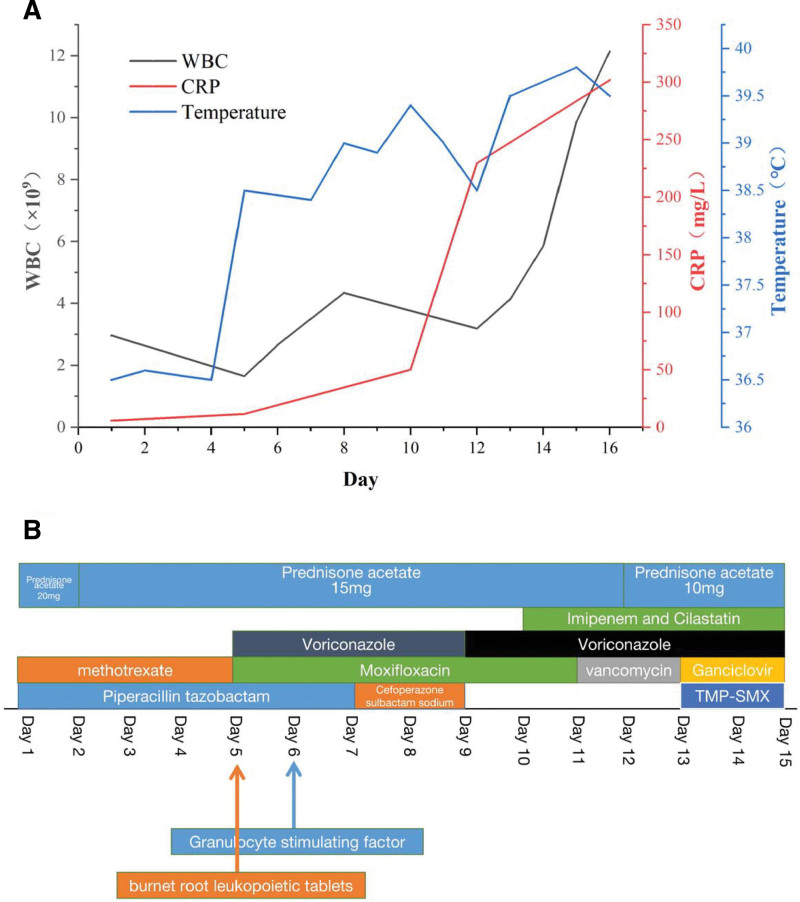
(A) Dynamic changes of white blood cell, C-reaction protein and temperature after the treatment, and (B) Clinical course of the patient.

**Figure 2.**
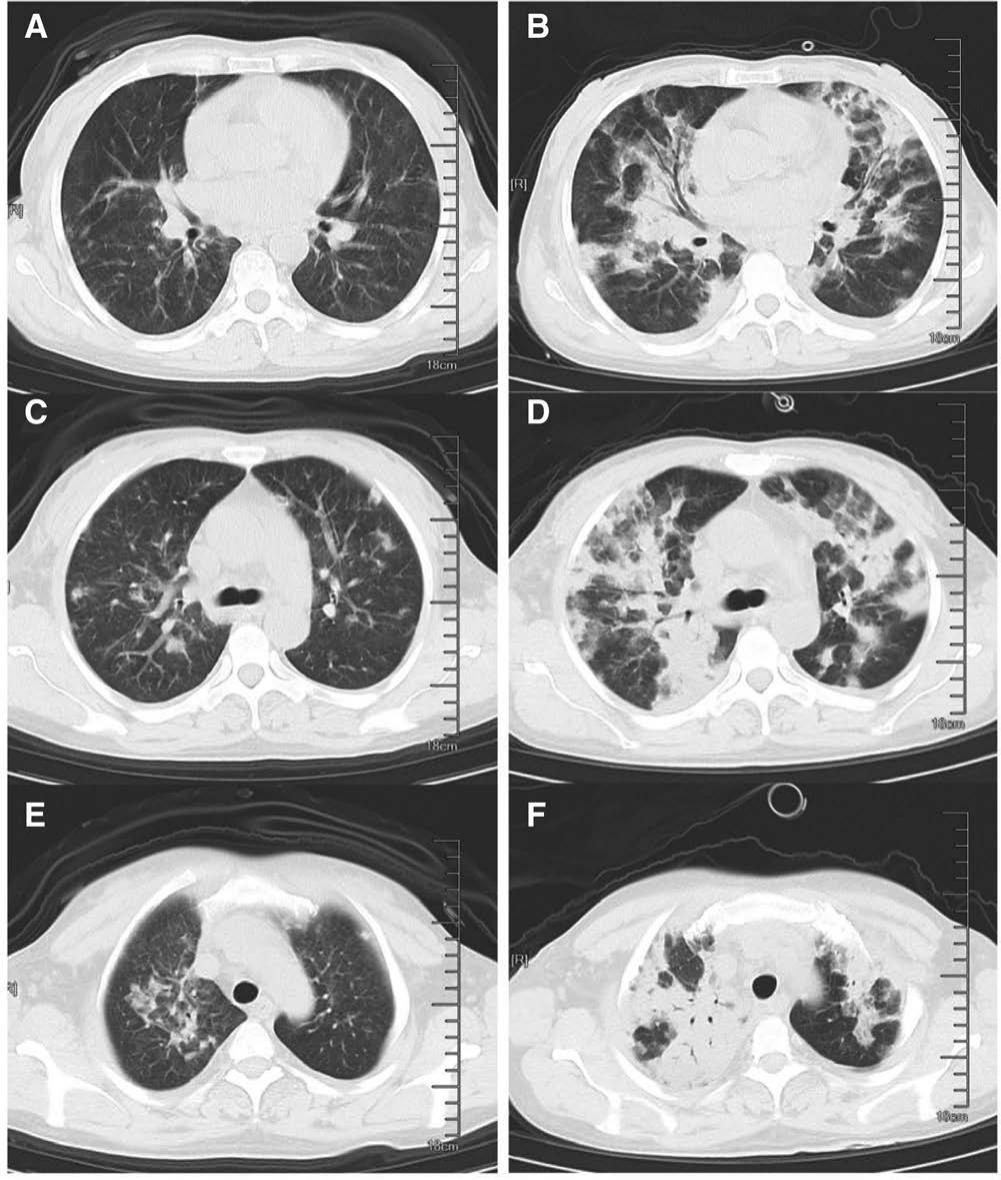
Chest CT-scan on the day 5 (A, C, E) and day 9, (B, D, F) of admission. Bilateral lung exudation and the range was significantly larger and the focus was denser than before. CT = computerized tomography.

Piperacillin/tazobactam sodium (4.5 g, every 12 hours) were prescribed to anti-infection empirically and taking prednisone acetate (20 mg/day) and methotrexate (2.5 mg/week) to control ANCA associated vasculitis (Fig. 1). On day 3, the patient’s cough symptoms worsened and did not shivering and fever. prednisone acetate reduced from 20 mg/day to 10 mg/day because of expansion of lung infection. On day 5, the patient had a sudden fever, with a maximum temperature of 38.5°C. Hypersensitive C-reactive protein increased to 11.4 mg/ L and procalcitonin was 0.27 ng/mL (Fig. 1). Chest CT on admission showed bilateral lung fields are clear, multiple patchy density increasing shadows in both lungs with unclear boundary (Fig. 2). WBC had fallen to 1.65 × 10E9/L and burnet root leukopoietic tablets (A Chinese patent medicine that can increase white blood cells) was given. Piperacillin/tazobactam sodium was switched to Cefoperazone/sulbactam sodium (1.5 g, every 12 hours) and moxifloxacin (0.4 g/day) and oral fluconazole (200 mg/day) for the treatment of severe infection (Fig. 1). On day 6, The sputum analysis of acid-fast bacilli and fungi smears was negative and a large number of G + cocci and many G-bacilli were found in the sputum smear. Beta-D-glucan level was 27.9 pg/mL (<10 pg/mL) and bacterial endotoxin level was 0.03 EU/mL. The count of WBC rebounded to 2.66 × 10E9/L, recombinant human granulocyte stimulating factor injection was given. Oral methotrexate was stopped because of the severe infection. On day 8, the patient developed fever again, and the maximum temperature reached 39°C. Chest CT performed on day 9 showed multiple patchy density increasing shadows in both lungs and the range was larger than 4 days before (Fig. 2). On day 11, the temperature of the patient reached to 39.4°C, and oxygen saturation was 85% without Oxygen inhalation and 92% to 96% with the therapy of high-flow nasal cannula (HFNC). Cefoperazone/sulbactam sodium and moxifloxacin were switched to Imipenem/cilastatin (1 g, every 12 hours). Oral Fluconazole was switched to Intravenous voriconazole (0.1 g, every 12 hours). On day 12, the patient was transferred to intensive care unit. Chest CT performed and showed bilateral lung exudation and the range was significantly larger and the focus was denser than 4 days before. Oxygen saturation could only be maintained at 85% with HFNC, so she discontinued HFNC and be treated with high-flow mask oxygen inhalation (10 L/minute). Vancomycin (500 mg, every 8 hours) was added for the treatment of severe infection. On day 13, the oxygen saturation decreased to about 85% with the treatment of noninvasive ventilator, and the partial pressure of oxygen dropped to 51.8 mm Hg. C-reactive protein (CRP) rapidly increased to 247.6 mg/L (Fig. 1). She refused to intubate and blood filtration was performed to remove toxins from her body in the next 3 days. On day 14, m next generation sequencing detected human alphaherpesvirus 1 with 9 high-confidence sequence reads (64.29% relative abundance) and Pneumocystis jirovecii with 61 high-confidence sequence (69.32% relative abundance) reads in blood. Ganciclovir (0.25g every 12 hours) was synergistically used to anti-human alphaherpesvirus 1 and trimethoprim-sulfamethoxazole (TMP-SMX) (240/ 1200 mg, every 8 hours) was used to treat PJP. On days 16, the patient died of respiratory failure.

## 3. Discussion

The relationship of hemodialysis with PJP infection has not been specifically stressed previously. Data on occurrence and risk factors for PCP in patients with hemodialysis are sparse. An observational study from Denmark showed that the incidence rates of first-time hospitalization for PCP was 30 times higher in patients with hemodialysis (43.1 per 100,000 person-years of follow up) compared to the population controls (1.43 per 100,000 person-years of follow up).^[5]^ To further summarize the clinical characteristics of PCP in hemodialysis patients, we searched the literature data base and 4 previous cases have been found (Table 2). Although the details of diagnosis and treatment of hemodialysis patients with PCP were very limited in those previous literatures, interestingly, all patients received long-term immunosuppressive therapy and 80% of patients died, which was significantly higher than the mortality in previous observational studies (43.8% in hospitalized children in Africa,^[10]^ 14.3% in human immunodeficiency virus (HIV)-infected patients in Europe,^[11]^ 44.2% in non- HIV immunocompromised patients in multiple countries,^[12]^ and 45.1% in HIV-negative patients in Asia).

**Table 2:** Case series of hemodialysis patients with PCP.

Case	Age/gender	Medical history	Immunosuppressant	Outcome
1^[6]^	25/Female	Heart transplant, distal pancreatectomy, uremia	Unknow	Alveolar proteinosis in recover period, alive
2^[7]^	23/Female	systemic lupus erythematosus (SLE), type B insulin resistance syndrome, uremia	Prednisolone	Died of pneumocystis jiroveci pneumonia
3^[8]^	62/Female	Anti-glomerular basement membranous disease, anti-neutrophil cytoplasmic antibodies (ANCA)- associated vasculitis, uremia	Corticosteroid	Died of systemic invasive aspergillosis
4^[9]^	34/Male	Granulomatosis with polyangiitis, uremia	Glucocorticoid cyclophosphamide	Died
5 (This case)	50/Female	Anti-neutrophil cytoplasmic antibodies (ANCA)- associated vasculitis, anti-glomerular basement membranous disease Hypothyroidism, uremia	Prednisone acetate methotrexate	Died of pneumocystis jiroveci pneumonia

PCP = pneumocystis pneumonia.

The key role of CD4 + T cells in resisting Pneumocystis infection has become a consensus.^[13]^ The most convictive evident is the strong correlation between the declining number of peripheral blood CD4 + T cell in HIV-infected patients and the increased risk of development of PCP,^[14]^ and a similar correlation also existed in non-HIV-infected patients who receiving immunosuppressive therapy.^[15]^ Shellito et al^[16]^ showed that T helper (Th) 1 and 2 LYM were recruited to the lung of mice with Pneumocystis infection. Th1 secretes IFN-γto activate alveolar macrophages, which are the primary cell type responsible for uptake and killing of Pneumocystis.^[13]^ The importance of B cells in body defense of Pneumocystis infection also has been proved by the animal models of B cell deficiencies^[17]^ and cases reported following B cell-targeted chemotherapy.^[18,19]^ However, in hemodialysis patients, the polarization of Th1 toward Th2 and the configuration of cytokine network depresses the cell mediated immunity.^[20]^ What is more, end-stage renal disease and renal replacement therapy premature immunological aging of T cell system^[21]^ and increase T cell turnover and apoptosis that bringing about depletion of naïve and central memory CD4 + and CD8 + T LYM ^[22]^ may underlie uremia-associated immune dysfunction. Patients with renal failure exhibit a general expansion of circulating monocytes^[23]^ with spontaneous activation that decreases the phagocytic capacity of monocytes^[24,25]^ and B cell deficiency and dysfunction that mediated by increased apoptosis of B cell^[26]^ and impaired differentiation and maturation of Transitional B cell.^[27]^ Under the combined effect of renal replacement therapy and end-stage renal disease, the immune function of anti-Pneumocystis in hemodialysis patients was seriously disabled. Therefore, it seems reasonable that hemodialysis patients have higher risk of infection with Pneumocystis jiroveci and worse prognosis.

In 2 of previous cases of deaths, both of patients had a medical history of AAV.^[8,9]^ Patients with collagen vascular disease have an elevated risk for PCP,^[28]^ particularly those with granulomatosis with polyangiitis,^[29]^ which was consistent with what we have observed. The younger male patient was considered to have AAV progression concurrently occurred during hospitalization for PCP, which could be proved by the relief of clinical symptoms after plasma exchange and the titer of pANCA.^[9]^ In our case, previous serological tests of the patients revealed a double-positive titer for MPO-ANCA and anti-GBM antibody. She had received immunosuppressive therapy for 6 months and the serological tests still showed a positive titer for pANCA in recent hospitalization. A large cohort study indicated that double-positive patients have higher frequency of alveolar hemorrhage and similar rate of relapse compared with single-positive AAV patients.^[30]^ Therefore, we cannot ignore the possibility that the Pneumocystis pneumonia and AAV lung injury coexist for her poor prognosis and outcome. Clinically, infections may have obscured AAV symptoms. Ground glass opacity was the most common major finding on chest CT of AAV patients, followed by honeycomb, reticular shadow, and nodule.^[31]^ On the other hand, a retrospective study aimed at non-HIV immunocompromised patients showed that the most frequent CT finding of PCP was symmetric, apically distributed ground glass opacity with peripheral sparing, followed by mosaic pattern infiltrate, consolidation, and architectural distortion,^[32]^ which consistent with chest CT of our patient. In our patient, she had hemoptysis at the first diagnosis of AAV, and the titer of pANCA showed 1:32 in recent hospitalization. Her glucocorticoid was reduced due to severe infection and then she had high fever, CRP increased rapidly, hemoglobin decreased, and her pulmonary symptoms were significantly aggravated. Since the titer of ANCA was not reviewed dynamically, it was hard for us to differentiate lung injury of AAV from PCP. The titer of ANCA is related to the disease condition, but it cannot be used as the main indicator to judge the disease activity. Erythrocyte sedimentation rate and CRP are closely related to the clinical condition of AAV, but they lack specificity, especially in AAV combined with infectious diseases. At present, the Birmingham vasculitis activity score scoring system is internationally recognized as the clinical index used to judge the systemic disease activity of vasculitis.^[33]^ This scoring system scores the whole-body performance, skin, mucosa, respiratory system, cardiovascular system, ear, nose, and throat system, digestive system, etc, and judges the degree of disease activity through the score.^[34]^ In summary, for patients with severe lung infections who have medical history of AAV, dynamic assessment of AAV activity during hospitalization should be performed to determine whether the pulmonary symptom is complicated with AAV lung injury, especially for those who have been treated with sufficient broad-spectrum antibiotics and empirical antifungal therapy but received unsatisfied treatment effect or failed to identify the pathogen after multiple etiological tests.

In our case, fluconazole and voriconazole were used as empirical therapy after the use of broad-spectrum antibiotics, immunosuppressant reduction, and symptomatic treatment, which followed by further deterioration of symptoms. Pneumocystis differs from most other fungi in that its cell membrane is rich in cholesterol but not ergosterol. Therefore, the use of azole drugs targeting ergosterol is ineffective against Pneumocystis infection. TMP-SMX is the preferred drug for the treatment of PCP because of its advantages of low cost, high susceptibility, and bioavailability in both intravenous and oral formulations.^[35]^ TMX-SMX is a combination of 2 antimicrobials that inhibit the folic acid metabolic pathway together on diverse targets.^[2]^ Its effectiveness in non-HIV-infected population has been proved in previous studies.^[36,37]^ The recommended strength for treatment in non-HIV-infected population is 15 to 20 mg/kg/day (based on the TMP component), divided into 3 to 4 doses per day, for 14 to 21 days.^[38]^ However, there is little evidence behind this recommendation in non-HIV populations. In our case, we gave TMP-SMX to against pneumocystis immediately after the next generation sequencing results reported, unfortunately, she died of severe pulmonary failure 3 days after taking the drug.

In consideration of her deteriorating immunity, we reduced the dose of glucocorticoids after she was hospitalized. However, what followed were high fever, progressive increase of C-reaction protein, decrease of leukocytes, which meant aggravation of her symptoms of infection. Antimicrobial degradation of PJP may make the lung function deteriorate sharply by producing a severe inflammatory response, theoretically, corticosteroids could suppress such reactions. However, after reviewing the literature, we surprisedly found that the role of glucocorticoids in the treatment of PCP is controversial. The use of adjunctive corticosteroid therapy (ACT) to improve the outcome of moderate-to-severe PCP in patients with HIV has become a consensus. Its effectiveness has been confirmed by several convincing randomized controlled trails in the last century.^[39–41]^ Glucocorticoids also have good performance in the rescue of severe HIV patients with PCP^[42]^ and the good long-term safety of ACT in HIV patients has also been proved.^[43]^ In contrast, there is no consensus on the role of glucocorticoids in non-HIV-infected patients. Four observational studies^[44–47]^ and 1 meta analysis^[48]^ indicated that ACT does not improve the survival of non-HIV-infected patients as has been described for HIV-infected patients with severe PCP. Three studies^[49–51]^ showed that only severe non-HIV patients with PCP can benefit from ACT and 1 studies^[52]^ demonstrated that high-dose glucocorticoids therapy were associated with increased mortality in non-HIV patients with PCP. Therefore, we believed that it was unwise that reducing or eliminating glucocorticoid therapy for non-HIV patients with PCP.

The prevalence of pulmonary colonization with pneumocystis jiroveci in hemodialysis patients was higher than healthy population,^[53]^ and the prognosis of hemodialysis patients with PCP seem to worser than non-hemodialysis patients. So, the prevention of PCP in hemodialysis patients should also be concerned. Unfortunately, there was no literature illuminating the risk factors of PCP in hemodialysis patients and prevention programs. Previous observational studies showed that high-dose and long-term glucocorticoid therapy and low LYMP count in non-HIV and non-organ transplant patients were highly associated with PCP.^[54–58]^ A study showed a decrease in the percentage of CD4+, CD4+/CD8 + ratios and NK cell in patients with Cushing’s syndrome compared to controls.^[59]^ The stimulating signals from dendritic cells (DCs) are the beginning of T cell mediated adaptive immune response. Pharmacological dose of glucocorticoids attenuate DCs activity by inhibiting DCs maturation and downregulating the expression of major histocompatibility complex II and co stimulatory molecules that preventing the activation, proliferation and differentiation of naïve T cells.^[60]^ It explains why long-term and high-dose of glucocorticoids therapy are usually accompanied by a decrease in LYMP count. Therefore, at this stage, early recognization of PCP risk factors in hemodialysis patients such as monitoring blood CD4 + Tcell and CD4/CD8 ratio is of great significant. The use of TMP-SMZ when hemodialysis patients are suspected of fungal infection may be an reasonable measure to improve the prognosis of PCP.

## 4. Conclusion

The risk of PCP in hemodialysis patients may be higher than that in ordinary population, and the prognosis of patients with immunosuppression may be worse. Dynamic assessment of vasculitis activity is necessary for hemodialysis patients with AAV because infections may obscure lung symptoms of AAV. It is not recommended that hemodialysis patients with long-term immunosuppression should reduce or stop the dosage of immunosuppressive drugs during the treatment because it may aggravate the condition of PCP. There is still no clear conclusion on whether hemodialysis patients need preventive medicine, but the identification of risk factors and early diagnosis and treatment are important for improving the prognosis of PCP on hemodialysis population.

## Acknowledgements

The authors would like to thank the family of the patient for granting us permission to publish the case report.

## Author contributions

**Conceptualization:** Jingda Huang, Fang Zeng, Meirong Shen, Qiao Shu, Dehui Liu.

**Data curation:** Fang Zeng, Wang Xu, Meirong Shen.

**Writing – original draft:** Jingda Huang, Fang Zeng, Jiajie Li.

**Writing – review & editing:** Qiao Shu, Dehui Liu.
